# RUNX3-activated apelin signaling inhibits cell proliferation and fibrosis in diabetic nephropathy by regulation of the SIRT1/FOXO pathway

**DOI:** 10.1186/s13098-024-01393-x

**Published:** 2024-07-17

**Authors:** Xin Zhong, Jun Zhang

**Affiliations:** 1 Department of Nephrology, The Second Clinical Medical College), Zhujiang Hospital of Southern Medical University, No. 253, Middle Industrial Avenue, Haizhu District, Guangzhou, 510280 Guangdong Province People’s Republic of China; 2 Department of Nephrology, Longgang Central Hospital of Shenzhen, Shenzhen, Guangdong Province People’s Republic of China

**Keywords:** Diabetic nephropathy, RUNX3, Apelin, SIRT1/FOXO, Fibrosis

## Abstract

**Background:**

Diabetic nephropathy is a major secondary cause of end-stage renal disease. Apelin plays an important role in the development of DN. Understanding the exact mechanism of Apelin can help expand the means of treating DN.

**Methods:**

Male C57BL/6 mice was used and STZ treatment was implemented for DN model establishment. Lentivirus systems including Lv-sh-RUNX3 and Lv-Apelin were obtained to knockdown RUNX3 and overexpress Apelin, respectively. A total of 36 mice were divided into 6 groups (*n* = 6 in each group): control, DN, DN + LV-Vector, DN + Lv-Apelin, DN + LV-Apelin + LV-sh-NC and DN + Lv-Apelin + Lv-sh-RUNX3 group. In vitro studies were performed using mesangial cells. Cell viability and proliferation was assessed through CCK8 and EDU analysis. Hematoxylin and eosin staining as well as Masson staining was implemented for histological evaluation. RT-qPCR was conducted for measuring relative mRNA levels, and protein expression was detected by western blotting. The interaction between SIRT1 and FOXO were verified by co-immunoprecipitations, and relations between RUNX3 and Apelin were demonstrated by dual luciferase report and chromatin immunoprecipitation.

**Results:**

The DN group exhibited significantly lower Apelin expression compared to control (*p* < 0.05). Apelin overexpression markedly improved blood glucose, renal function indicators, ameliorated renal fibrosis and reduced fibrotic factor expression (*p* < 0.05) in the DN group, accompanied by elevated sirt1 levels and diminished acetylated FOXO1/FOXO3a (*p* < 0.05). However, RUNX3 knockdown combined with Apelin overexpression abrogated these beneficial effects, leading to impaired renal function, exacerbated fibrosis, increased fibrotic factor expression and acetylated FOXO1/FOXO3a versus Apelin overexpression alone (*p* < 0.05). In mesangial cells under high glucose, Apelin overexpression significantly inhibited cell proliferation and fibrotic factor production (*p* < 0.05). Conversely, RUNX3 interference enhanced cell proliferation and the secretion of fibrotic factors. (*p* < 0.05). Remarkably, combining Apelin overexpression with RUNX3 interference reversed the proliferation and fibrosis induced by RUNX3 interference (*p* < 0.05). Mechanistic studies revealed RUNX3 binds to the Apelin promoter, with the 467–489 bp site1 as the primary binding region, and SIRT1 physically interacts with FOXO1 and FOXO3a in mesangial cells.

**Conclusion:**

RUNX3 activated Apelin and regulated the SIRT1/FOXO signaling pathway, resulting in the suppressed cell proliferation and fibrosis in diabetic nephropathy. Apelin is a promising endogenous therapeutic target for anti-renal injury and anti-fibrosis in diabetic nephropathy. RUNX3 may serve as an endogenous intervention target for diseases related to Apelin deficiency.

**Supplementary Information:**

The online version contains supplementary material available at 10.1186/s13098-024-01393-x.

## Highlight

1. Apelin was significant downregulated in DN.

2. Apelin overexpression attenuates progression of DN.

3. Apelin inhibits mesangial cell proliferation and fibrosis via SIRT1/FOXO pathway.

4. RUNX3 transcriptionally upregulates Apelin expression.

5. RUNX3/Apelin axis regulates SIRT1/FOXO, mitigating DN progression.

**Supplementary Information:**

The online version contains supplementary material available at 10.1186/s13098-024-01393-x.

## Introduction

Diabetic nephropathy (DN) is a severe microvascular complication of diabetes mellitus, contributing to enhanced morbidity and mortality, and ultimately resulting in end-stage kidney disease (ESKD) [1]. The global prevalence of DN has increased dramatically in recent years, with an estimated 40% of patients with diabetes developing nephropathy [2, 3]. High glucose-stimulated mesangial cell proliferation and extracellular matrix (ECM) secretion and deposition can lead to mesangial expansion, which is an early characteristic pathological change in DN. This mesangial expansion is considered a primary cause of capillary lumen occlusion and glomerulosclerosis [4, 5]. Hyperglycemia, oxidative stress, inflammation and other factors are involved in the pathogenesis of DN. Traditional treatments to control blood glucose, blood pressure and proteinuria and new drugs such as SGLT-2 inhibitors and new non-steroidal mineralocorticoid antagonists have limited effects on inhibiting DN progression [6]. Current treatments are not sufficient to completely prevent the risk of diabetic nephropathy progressing to ESKD [7, 8]. Therefore, to deepen the study of the pathogenesis of diabetic nephropathy, new effective treatment strategy for promoting DN has a key role. In the treatment of anti-nephrosclerosis and renal fibrosis, targeted drugs that previously inhibit the pro-fibrotic factor TNF-β1 have poor anti-renal fibrosis treatment effect. Some experts have proposed that restoring the balance between pro-fibrotic and anti-fibrotic signaling pathways by targeted strengthening of endogenous anti-fibrotic molecules can be considered as a new anti-renal fibrosis strategy [9]. The exploration of endogenous anti-fibrotic molecules has become a direction of DN research.

Apelin is an endogenous ligand of the Apelin receptor located in the G protein-coupled receptor angiotensin structural domain [10]. Encoded by the APLN gene in the chromosomal region Xq25-26.1, Apelin is generated primarily by white adipose tissue, but it is also found in multiple tissues, which include the endothelial cells, vascular system, and kidney [11]. The in vivo existence of Apelin in different isoforms (Apelin-36, Apelin-17, and Apelin-13) by endoplasmic reticulum shearing has been observed [12]. Apelin, as an adipokine, can promote insulin secretion, increase insulin sensitivity, and improve insulin resistance [13, 14]. A clinical study showed that type 2 diabetes and DN were related with decreased serum Apelin levels [15]. However, another research revealed that Apelin levels in diabetics were elevated [16]. Apelin-13 could suppress the transforming growth factor-β1 (TGF-β1) expression, which belongs to a critical mediator of renal fibrosis, thus alleviate kidney tissue fibrosis [17]. Previous studies have also shown that Apelin exhibits anti-inflammatory, anti-podocyte apoptosis, and anti-fibrotic effects. Apelin is a promising therapeutic target for DN. However, the specific mechanisms of Apelin’s actions in DN kidney tissues and cells warrant further in-depth investigation.

Apelin can activate SIRT1 that subsequently regulates downstream pathways. SIRT1, a NAD + dependent deacetylase, is involved in multiple cellular pathways by deacetylating proteins [18]. Specifically, SIRT1 is implicated in the treatment of fibrosis, diabetes, cancer, and aging by modulating the activity of several transcription factors such as p53, TGF-β, YY1, and FOXO through deacetylation [19]. FOXO proteins function primarily as transcription factors in the nucleus and bind to the FOXO binding consensus domain of target genes, regulating the expression of these genes. The transcriptional activity of FOXO is regulated by post-translational modification, including phosphorylation, acetylation, and ubiquitination, which determine subcellular localization, binding with DNA or other regulatory factors, and degradation, among other features [20]. SIRT1 regulates the acetylation of FOXO family members, including FOXO1, FOXO2, FOXO3a, FOXO4, and FOXO6, to repress their processes that involve in the transcription of other downstream genes [21]. It is worth noting that FOXO signaling participates in multiple biological pathways, which included cell cycle regulation, oxidative response, metabolism, and aging. A recent study reported that FOXO signaling pathway was enriched and activated in DN [22]. The acetylation of FOXO family was also demonstrated to be related with the progression of diabetic cardiomyopathy [23]. Nevertheless, the impact of Apelin on the FOXO signaling pathway remains unclear and requires further investigation.

Runt-related transcription factors (RUNX) proteins 1, 2 and 3 (RUNX1, RUNX2 and RUNX3) are crucial in binding to DNA by the combination of the Runt homology domain (RHD) with promoters and enhancers [24]. RUNX3 can regulate several downstream genes related with diverse biological processes that comprise cell differentiation, migration, and death, and these target genes may facilitate the progression of fibrosis. RUNX3 is reported to interact with other transcription factors, including p53 and TGF-β, which are involved in fibrosis and other pathological conditions [25, 26]. For instance, RUNX3 deficiency in dendritic cells has been shown to cause skin fibrosis and enhanced the severity regarding skin inflammatory status and fibrosis in bleomycin-induced mice [27]. Additionally, recent studies have suggested that RUNX3 could interact with various signaling pathways, for example, the Wnt/β-catenin signaling, which is implicated in various diseases including fibrosis and cancer [28]. Despite these findings, the curative effect of RUNX3 in DN complications remains inadequately investigated, and the underlying molecular mechanism has not been deeply studied. Whether RUNX3 can transcriptionally activate Apelin has not been studied in the past. Further research is required for exploring therapeutic potential of RUNX3 and its role in DN.

The present study hypothesized that the transcription factor RUNX3 could activate Apelin transcription by binding to the Apelin promoter region. This, in turn, could lead to the upregulation of SIRT1 expression and the suppression of FOXO’s transcriptional activity. The regulation role of RUNX3 on SIRT1/FOXO signal pathway was also firstly investigated in DN. This study helps to clarify the upstream and downstream regulatory mechanisms of Apelin in renal fibrosis associated with DN, providing a theoretical basis for Apelin as an endogenous anti-fibrotic therapeutic target for DN.

## Methods

### Animal models construction and lentivirus treatment

According to previous studies [29, 30], mice models were constructed, and lentivirus transfection was performed. Briefly, a total of 36 adult male C57BL/6 mice (7–8 weeks, 22 ± 2 g weight) were obtained (SLAC, Shanghai, China), and were kept in a temperature-constant environment (22 ± 1 °C) with a 12 h-light/dark cycle and 50 ± 10% humidity. All animal experiments were conducted in accordance with the guidelines provided in the Guide for the Care and Use of Laboratory Animals of the Chinese National Institutes of Health. The mice were randomly allocated into 2 groups: The mice were randomly allocated into 2 groups: control group (*n* = 6) and DN group (*n* = 30). Then, the 30 of mice with DN were divided into 5 groups (*n* = 6 in each group) for subsequent gene transfection treatments. To induce DN, the mice were intraperitoneally injected with 50 mg/kg STZ (S0130, Sigma, St. Louis, MO, USA) dissolved in 100 mM citrate buffer (pH 4.5) for 5 consecutive days. The control group was injected with an equal volume of citrate buffer. Blood glucose, 24-hour proteinuria, serum creatinine, and blood urea nitrogen were measured after 28 weeks. The blood glucose concentration higher than 16.7 mmol/L was confirmed as diabetes, and 24-hour proteinuria higher than 30 mg was verified as DN. Subsequently, all animals were euthanized using a carbon dioxide release device, and kidney tissues were collected.

Gene overexpression or knockdown models were established in vivo using lentivirus (Lv) systems that were constructed by Genechem Technology Co., Ltd., Shanghai, China. Lv-sh-RUNX3 and Lv-Apelin were obtained to knockdown RUNX3 and overexpress Apelin, respectively, while Lv-sh-NC (randomly scrambled) or Lv-vector was used as the negative control. Then 30 of DN mice were divided into 5 groups: DN, DN + LV-Vector, DN + Lv-Apelin, DN + LV-Apelin + LV-sh-NC and DN + Lv-Apelin + Lv-sh-RUNX3. Then, DN mice were anesthetized by 1% pentobarbital sodium (40 mg/kg), and their kidneys were exposed via a back incision. A total of 100 µL of lentiviral vector solution (2 × 10^6^ IU/kidney) was inoculated into mice through injection into the kidney parenchyma (6 mice for each transfection group), after which the incisions were closed. After 4 weeks, all animals were euthanized by cervical dislocation, and kidney tissues were collected.

### Immunofluorescence

Kidney tissue sections were incubated with 4% polyformaldehyde for 15 min at room temperature. Subsequently, sections were incubated with the blocking buffer containing 5% goat serum for 1 h. After washed by PBS for 3 times, sections were cultured in the diluted primary antibody overnight at 4℃. Then, sections were incubated with the fluorescent labeled secondary antibody for 1 h at room temperature, protected from light, after washed by PBS. Finally, sections were stained with DAPI and sealed with sealing solution containing anti fluorescence quenching agent. The expressions of Apelin, α-SMA, fibronectin, collagen I, RUNX3 and SIRT1 were observed under a fluorescence microscope (Olympus, Japan). Antibodies included Apelin (GTX37465, GeneTx, USA; 1:100), α-SMA (48,938, Cell signaling technology, USA; 1:100), fibronectin (ab2413, Abcam, UK; 1:200), collagen I (ab270993, Abcam, UK; 1:100), RUNX3 (13,089, Cell signaling technology, USA; 1: 400), SIRT1 (8469, Cell signaling technology, USA; 1:100) and secondary antibody (ab150077, 1:200; ab150113, 1:200; Abcam, UK).

### Cell culture and transfection

Mouse glomerular endothelial cells (MGECs), mesangial cells (MCs), glomerular podocytes cell line MPC5, and renal tubular epithelial cell line TCMK-1 were acquired from the American Type Culture Collection (Manassas, VA, USA). The cells were maintained in Dulbecco’s modified Eagle’s medium (DMEM, Gibco, MD, USA) supplemented with 10% fetal bovine serum, penicillin (100 U/mL), and streptomycin (100 µg/mL) in a humidified atmosphere with 5% CO2 at 37 °C. All prepared cells were firstly allocated into two groups: normal glucose (NG) and high glucose (HG). We obtained mannitol (Sigma, St. Louis, MO, USA) for controlling osmotic pressure in both groups. The NG group cells were treated with medium that contained 5.5 mM glucose and 19.5 mM mannitol (Sigma, St. Louis, MO, USA), while the medium in HG group were supplemented with 25 mM glucose [31].

For in vitro overexpression or knockdown models, vectors mediating overexpression of Apelin (OE-Apelin), overexpression of RUNX3 (OE-RUNX3), and knockdown of RUNX3 (sh-RUNX3) were obtained from Genechem Technology (Shanghai, China). The vectors transfection was conducted by Lipofectamine™ 3000 as per its instruction protocol (Invitrogen, Carlsbad, CA, USA). After 48 h of transfection, cells were cultured under high glucose conditions. For activation or inhibition of SIRT1, cells were treated with Sirt1 activator SRT1720 (50 mg/kg) or inhibitor EX527 (10 mg/kg) for 1 h. Subsequent experiments were preformed after incubation for an additional 48 h.

### Cell viability

The viability was assessed by the CCK8 kit (Solarbio, Beijing, China) as per instructions of kit. Briefly, cells were inoculated into a 96-well plate (density: 2 × 10^4^ cells per well) and incubated at 37 °C for 12 h. After treatment according to the experimental design, cells in each well were added with CCK8 reagent (10 µL) and incubated for 2 h. The absorbance (450 nm) was measured through a microplate reader (Bio-Rad, Hercules, CA, USA).

### Cell proliferation

The proliferation of cells was evaluated by EDU Cell Proliferation Kit (Beyotime, Shanghai, China). In brief, cells were inoculated into a 24-well plate (density: 1 × 10^5^ cells/well) and cultured at 37 °C overnight. Following this, each well was added with 50 µM EDU solution, incubated for 2 h and then fixed with 4% paraformaldehyde. Cell nuclei were then stained by DAPI, and the results were visualized using a fluorescence microscope (Olympus, Tokyo, Japan).

### Hematoxylin and eosin (H&E) staining and Masson staining

In brief, the kidney tissues were fixed with 4% paraformaldehyde, followed by dehydrating in an ascending ethanol series, and finally being embedded in paraffin. The 5-µm-thick sections were cut from the tissue block and dewaxed in xylene and rehydrated in a descending alcohol series. Subsequently, we implemented H&E or Masson assay kit (Solarbio, Beijing, China) for staining the sections as per the instructions of kit. Finally, samples were examined under a light microscope (Olympus, Tokyo, Japan).

### Real-time quantitative PCR (RT-qPCR)

The TRIzol reagent (Invitrogen, Carlsbad, CA, USA) was purchased for isolating total RNA. cDNA was synthesized according to the protocols of the reverse transcription reagent kit (TaKaRa, Tokyo, Japan). RT-qPCR was performed using SYBR Green qPCR Mix (TaKaRa) on an ABI quantitative PCR system (Waltham, MA, USA). β-actin was selected as an endogenous control, and the mRNA relative levels were calculated using the 2^−ΔΔCt^ method. The gene primers used in the study were listed as follows:

 Apelin-F: 5’- CGATGGGAATGGGCTGGAAGA − 3’.

 Apelin-R: 5’- CAGAAAGGCATGGGTCCCTTATG − 3’.

 PCNA-F: 5’- TGAAGCACCAAACCAGGAG − 3’

 PCNA-R: 5’- GAAGGCATCTTTACTACACAGC − 3’

 CyclinD1-F: 5’- GAGACCATCCCCCTGACGGC-3’.

 CyclinD1-R: 5’- TCTTCCTCCTCCTCGGCGGC-3’.

 RUNX3-F: 5’- CAGGTTCAACGACCTTCGATT − 3’

 RUNX3-R: 5’- GTGGTAGGTAGCCACTTGGG − 3’

 β-actin-F: 5’- AGGGGCCGGACTCGTCATACT − 3’.

 β-actin-R: 5’- GGCGGCACCACCATGTACCCT − 3’.

### Western blotting

The total proteins were extracted using RIPA lysis buffer (Bocai, Shanghai, China), and BCA Protein Assay Kit (Beyotime, Shanghai, China) was obtained for determining protein concentration of the supernatant. Protein separation was performed on 10% SDS-PAGE gels, followed by transfer of proteins onto PVDF membranes (Millipore, MA, USA). After blocking with 6% non-fat milk in Transfer Cell for 1 h at room temperature, the membranes were incubated with primary antibodies that included anti-Apelin (ab125213, 1:1000), anti-α-SMA (ab7817, 1:1000), anti-fibronectin (ab2413, 1:1000), anti-collagen I (ab260043, 1:1000), anti-FOXO1 (PA5-104560, 1:1000), anti-FOXO2 (ab23630, 1:1000), anti-FOXO3a (A256022, 1:1000), anti-FOXO4 (YK0078, 1:1000), anti-FOXO6 (PA5-106411, 1:1000), anti-SIRT1 (ab110304, 1:1000), anti-RUNX3 (ab135248, 1:1000) and anti-β-actin (ab8226, 1:1000), overnight at 4 °C. The membrane was then incubated with an HRP-conjugated rabbit anti-mouse IgG secondary antibody (ab6728, 1:1000) for 1 h at room temperature. Protein bands were visualized using a chemiluminescent HRP substrate and analyzed using the ImageJ software.

### Co-immunoprecipitations (COIP)

#### COIP

P procedure was conducted via the Pierce Co-Immunoprecipitation Kit (Thermo Fisher Scientific, Waltham, MA, USA), as per the protocols of manufacturer. Briefly, lysates of cells were isolated and maintained with anti-SIRT1 (ab110304, 1:1000) antibody at 4 °C for 12 h. Prior to this, magnetic beads were pre-treated and coupled with the secondary Rabbit Anti-Mouse IgG H&L (HRP) antibody (ab6728, 1:1000). Subsequently, 50 L of these antibody-coupled magnetic beads were added to the samples and incubated for 8 h at 4 °C. The mixture was boiled with SDS buffer, and the western blotting was conducted to verify the results, as described above.

### Dual luciferase report

In brief, the potential binding site of RUNX3 located in Apelin promoter was predicted using the bioinformatics tool JASPAR (https://jaspar.genereg.net/). The Apelin promoter sequence containing these sites was amplified by PCR and cloned into the pGL3 luciferase reporter vector (Nuopu Biotechnology, Shanghai, China V001746). The groups included: F1 (containing site2), F2 (containing site1), WT (unmutated), MUT-1 (site1 mutated), MUT-2 (site2 mutated), MUT-3 (site1 and site2 mutated). Co-transfection was performed using Lipofectamine 3000 (Invitrogen, Carlsbad, CA, USA) as per the protocols. After 24 h, luciferase activity was measured using a dual-luciferase reporter system (Beyotime, Shanghai, China, RG027).

### Chromatin immunoprecipitation (ChIP)

To verify the interaction between RUNX3 and different sites in the Apelin promoter region, the ChIP assay was carried out via the EZ-ChIP Chromatin Immunoprecipitation Kit (Millipore, MA, USA) as per manufacturer’s protocol. Briefly, approximately 1 × 10^6^ cells were cross-linked with formaldehyde (Sigma, St. Louis, MO, USA) for 15 min at 37 °C, and ultrasound was used to treat the samples for obtaining DNA fragments. The samples were then immunoprecipitated with magnetic beads that coupled with specific antibodies and anti-IgG for negative control. RT-qPCR was performed to analyze the precipitated DNA as described above.

### Nuclear cytoplasmic separation

Cells were lysed by Nuclear and Cytoplasmic Protein Extraction Kit (P0028, Beyotime, Shanghai, China), followed by the centrifugation at 12, 000 g for 5 min at 4℃. The supernatant contained the cytoplasm protein, and the precipitate was the nuclear protein. Then, FOXO1 and FOXO3a in nucleus and cytoplasm were detected by western blot. Histone 3 was used for the reference of the nuclear protein and actin was for the reference of the cytoplasm protein.

### Statistical analysis

All data were expressed as mean ± standard deviation (SD). GraphPad Prism 8 (La Jolla, CA, USA) was used for statistical analysis. Student’s t-test (two-tailed) was applied to compare two groups, and one-way ANOVA followed by Newman-Keuls post hoc test was used for comparison among multiple groups. Two-way repeated measures ANOVA was implemented to determine the differences between multiple groups and timepoints. Statistical significance was considered at *P* < 0.05.

## Results

### Apelin level was downregulated in STZ-treated DN mice

**Fig. 1:**
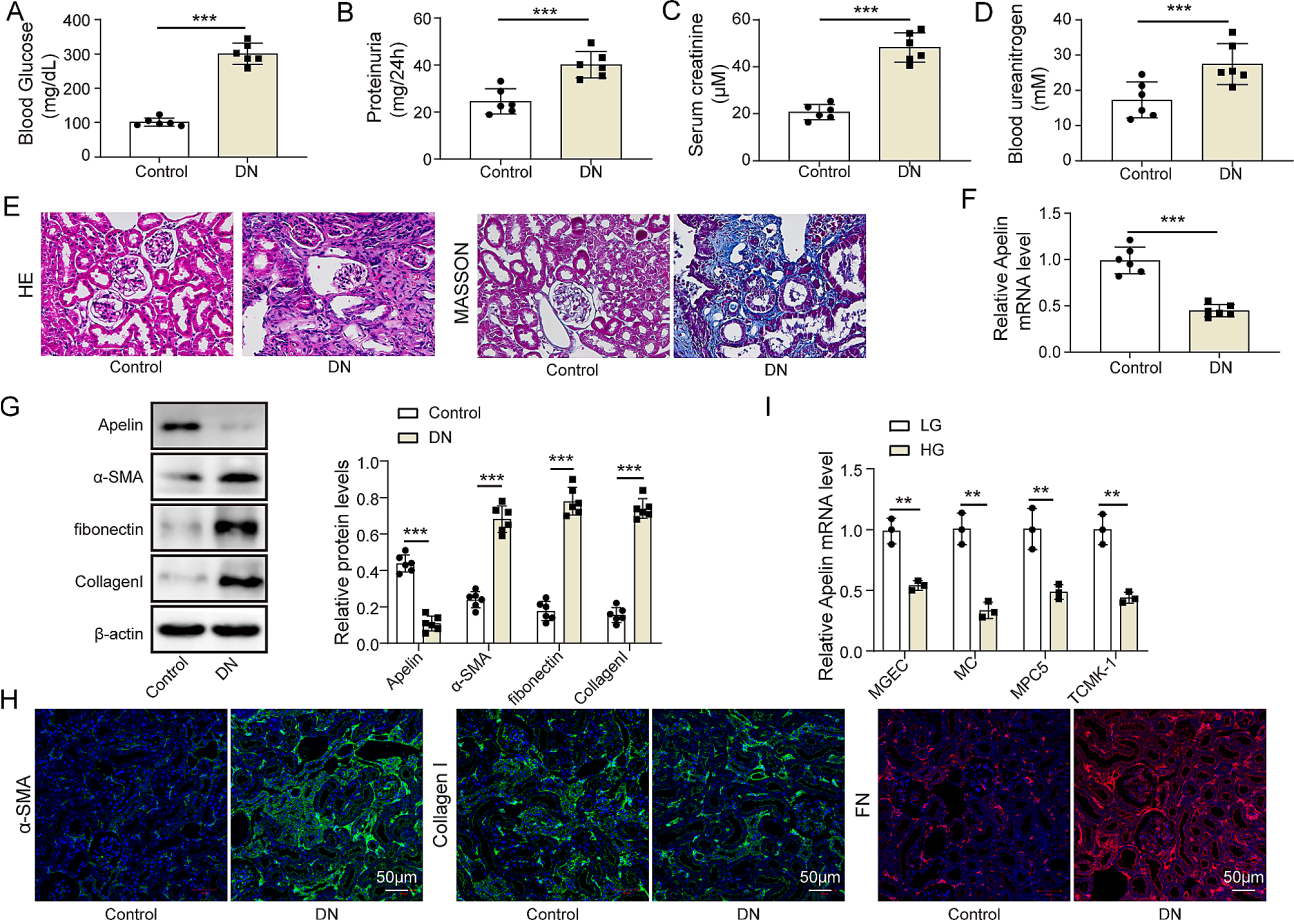
Apelin expression was downregulated in STZ-induced DN mice (*n* = 6 in each group). (**A**) The blood glucose level. (**B**) The 24-hour proteinuria level. (**C**) The serum creatinine level. (**D**) The blood urea nitrogen level. (**E**) The H&E and Masson staining revealed the kidney structural changes (400X). (**F**) The relative mRNA expression of Apelin detected by RT-qPCR. (**G**) The protein expression determined using western blotting. (**H**) The protein expression in tissues determined by immunofluorescence (scale: 50 μm; Red: FN; Green: α-SMA or Collagen I, ; Blue: nucleus). (**I**) The relative mRNA expression of Apelin in various cell lines treated by different concentration of glucose. *p < 0.05, **p < 0.01, ***p < 0.001. Diabetic nephropathy, DN; hematoxylin and eosin, H&E; high glucose, HG; mesangial cells, MCs; mouse glomerular endothelial cells, MGECs; normal glucose, NG; LG, low glucose

Blood glucose levels, renal function indicators, and renal histopathology were assessed in the DN group and control group (*n* = 6 per group) to evaluate the DN model. (Figure 1A and D). Compared to the control group, the DN group exhibited significantly elevated levels of blood glucose, 24-hour proteinuria, serum creatinine, and blood urea nitrogen, indicating renal impairment (*p* < 0.05). Furthermore, kidney structural differentiation was assessed using H&E and Masson staining, and results are presented in Fig. 1E. For control group, clear tubular structure and normal glomeruli could be found in kidney, Tubular epithelial cells were neatly arranged, and intact mesangial substrate membrane were observed. However, for DN group mice, the glomeruli were enlarged, as well as tubular lumen was dilated with irregularly thickened mesangial substrate membranes. In addition, marked vacuolization of tubular epithelial cells and infiltration of interstitial inflammatory cells were observed. The content of strong positive stained by Masson kit was also increased, suggesting marked interstitial fibrosis. Apelin levels were determined to investigate their expression in DN (Fig. 1F-G, Figure S1). The results indicated that Apelin levels in the DN mice kidney tissues was reduced (*p* < 0.05). Furthermore, western blotting assay results revealed increased levels of fibrotic factors including α-SMA, fibronectin, and collagen I in the DN mice kidney tissues (*p* < 0.05, Fig. 1G). The immunofluorescence results were consistent with the western blotting data, including the elevation in α-SMA, fibronectin, and collagen I (*p* < 0.05) (Fig. 1H). For investigating the role of glucose on Apelin level in different types of kidney cells, MGEC, MCs, MPC5, and TCMK-1 cells were cultured for 48 h (Fig. 1I). The results indicated that Apelin level was most decreased in MC at high glucose levels (*p* < 0.05). These findings indicated that Apelin expression was downregulated in the kidney tissues of DN mice, with the most significant decrease observed in MCs. This indicated that MCs exhibited the most significant regulation of Apelin in response to a high glucose environment. Therefore, MCs were selected as the research subject.

### Apelin overexpression inhibited proliferation and fibrosis in HG-treated MCs

**Fig. 2:**
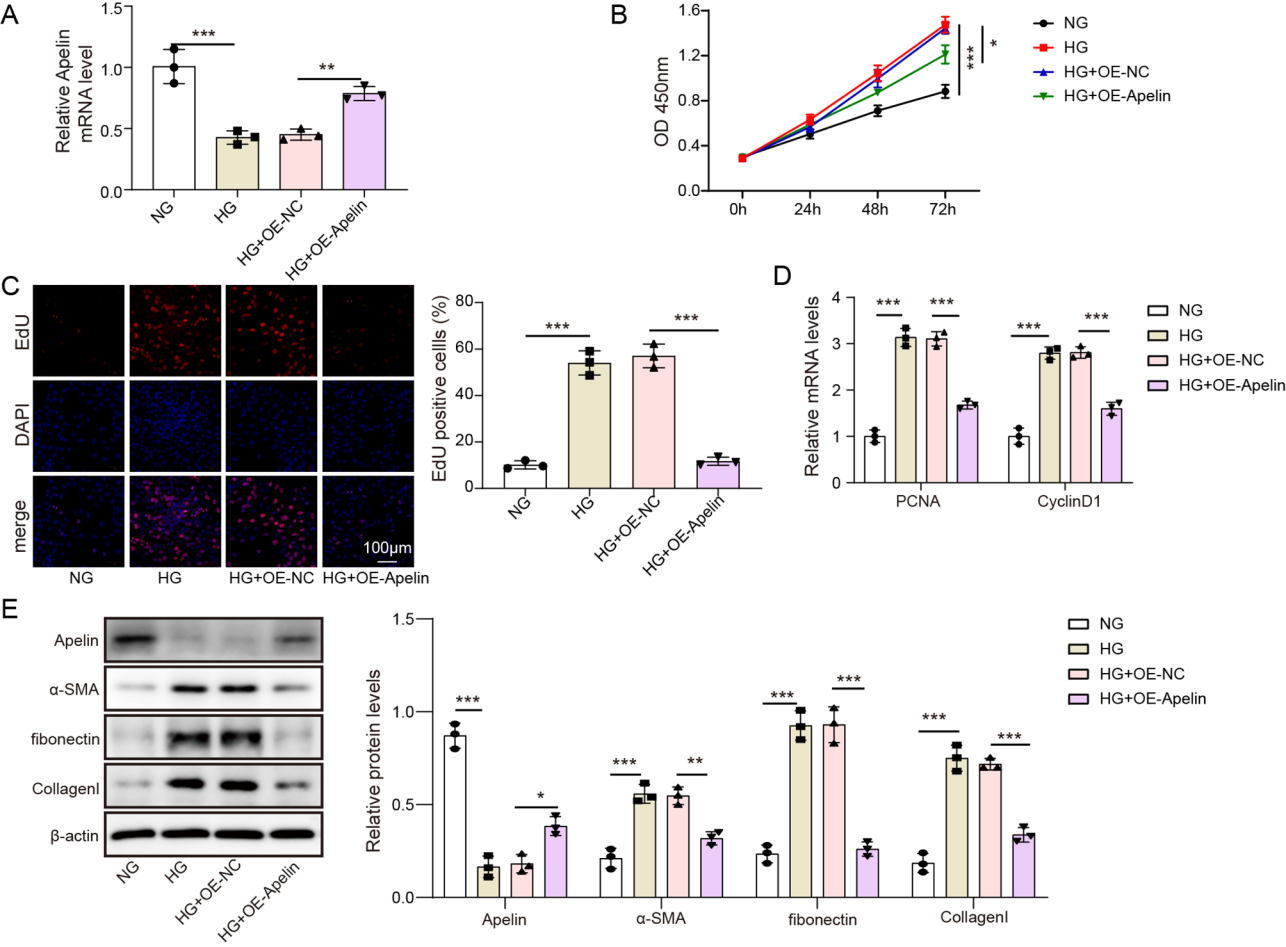
Apelin overexpression inhibited proliferation and fibrosis in HG-treated MCs. (**A**) The relative mRNA expression of Apelin detected by RT-qPCR. (**B**) Cell viability evaluated by CCK8 assay. (**C**) Cell proliferation determined using EDU staining. (**D**) The relative mRNA expression of PCNA and CyclinD1 detected by RT-qPCR. (**E**) The protein expression of Apelin, α-SMA, fibronectin, and collagen I determined using western blotting. *p < 0.05, **p < 0.01, ***p < 0.001. high glucose, HG; normal glucose, NG; overexpression, OE

To investigate the biological effects of Apelin in DN, an in vitro model was established by treating MCs with HG. Apelin overexpression or empty vector transfection were implemented on normal and HG-treated MCs, respectively. Apelin expression was significantly downregulated in the HG group compared to the normal control group. In the HG group transfected with Apelin overexpression vector, Apelin expression was markedly higher than in the HG empty vector control group, indicating successful overexpression of Apelin in the transfected cells. (*p* < 0.05, Fig. 2A). We also verified that transfecting Apelin vectors could upregulate its expression in normal glucose condition (FigureS2). Cell viability was determined in various group of MCs. As illustrated in Fig. 2B, cells treated by HG possessed higher survival rate compared to NG group, while Apelin overexpression in HG-treated cells eliminated this difference (*p* < 0.05). As presented in Fig. 2C, the number of EDU-positive cells in HG group was dramatically enhanced, whereas Apelin overexpression in HG-treated cells dramatically reduced the proportion of EDU-positive cells (*p* < 0.05). For measuring the levels of the proliferation markers PCNA and CyclinD1, RT-qPCR was employed. The results showed that PCNA and CyclinD1 levels were upregulated in HG group compared to NG group, and this trend was eliminated by overexpression of Apelin (*p* < 0.05, Fig. 2D). The protein expression of Apelin, α-SMA, fibronectin, and collagen I levels were monitored (Fig. 2E). Apelin expression was downregulated, while fibrotic factors including α-SMA, fibronectin, and collagen I levels were upregulated in HG-treated cells compared to NG-treated cells, while Apelin overexpression had reversed effects (*p* < 0.05).

### Apelin overexpression inhibited MCs proliferation and fibrosis through the SIRT1/FOXO signaling pathway

**Fig. 3:**
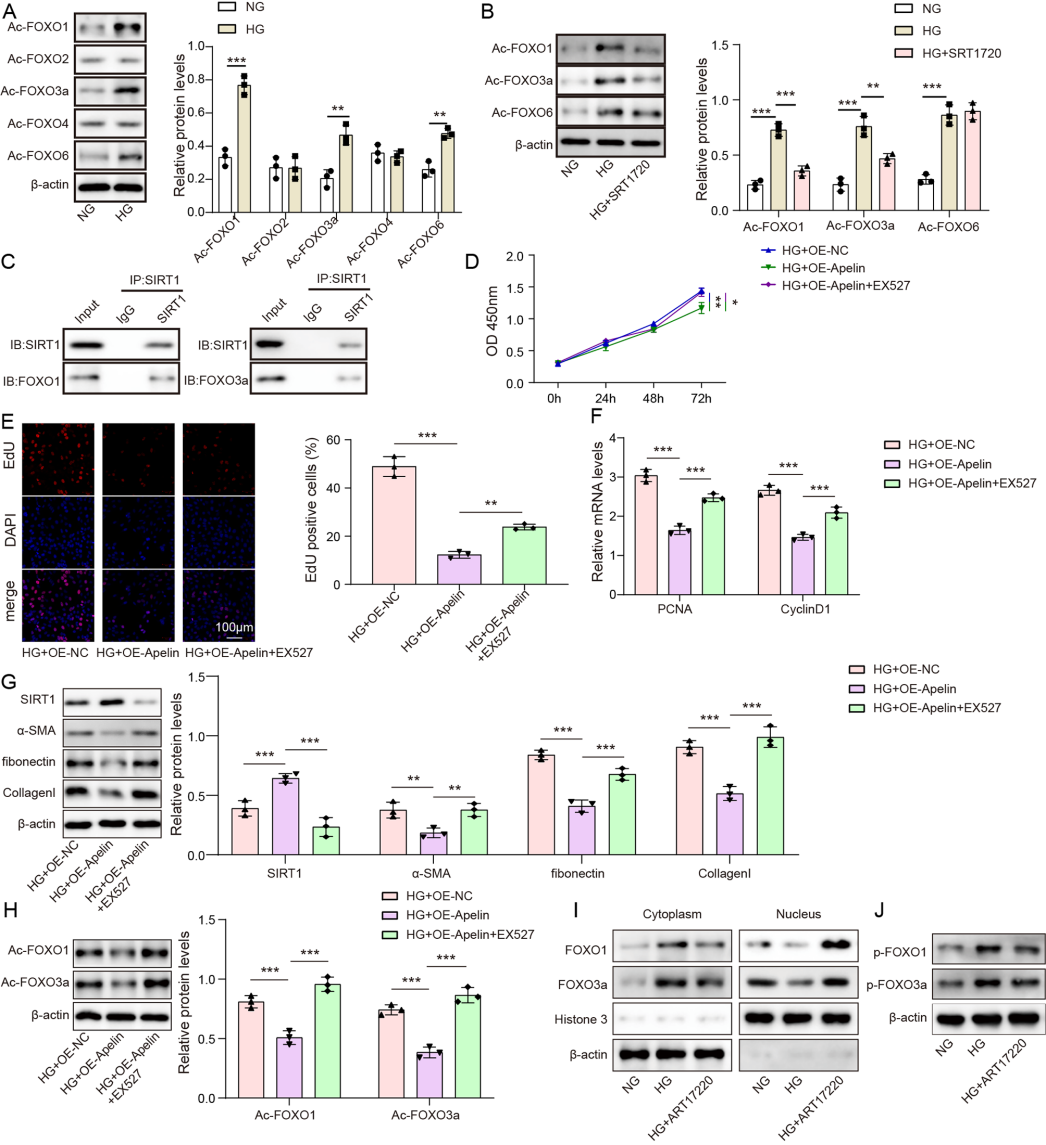
Apelin overexpression inhibited MCs proliferation and fibrosis through the SIRT1/FOXO signaling pathway. (**A**) The acetylation status of FOXO1, FOXO2, FOXO3a, FOXO4, and FOXO6 assessed by western blotting. (**B**) The effects of SIRT1 activation on acetylation status of FOXO1, FOXO3a, and FOXO6. (**C**) COIP assay proved the interactions between SIRT1 and FOXO1, and between SIRT1 and FOXO3a. (**D**) Cell viability evaluated by CCK8 assay. (**E**) Cell proliferation determined using EDU staining. (**F**) The relative mRNA expression of PCNA and CyclinD1 detected by RT-qPCR. (**G**) The protein expression of SIRT1, α-SMA, fibronectin, and collagen I determined using western blotting. (**H**) The acetylation status of FOXO1 and FOXO3a assessed by western blotting. (**I**) FOXO protein nuclear translocation. (**J**) FOXO phosphorylation. *p < 0.05, **p < 0.01, ***p < 0.001. high glucose: HG; normal glucose: NG; overexpression: OE

To understand the biological effects and interaction between SIRT1 and FOXO proteins in HG-induced MCs, the levels of the acetylation status of FOXO1, FOXO2, FOXO3a, FOXO4, and FOXO6 were assessed using western blotting (Fig. 3A). Immunoprecipitation experiments using acetyl lysine antibodies followed by western blotting with FOXO1, FOXO2, FOXO3a, FOXO4, and FOXO6 antibodies were performed (Fig. 3A). The HG group exhibited significantly higher levels of Ac-FOXO1, Ac-FOXO3a, and Ac-FOXO6 proteins (*p* < 0.05). SIRT1 is known to be a key factor in fibrosis as it regulates FOXO through deacetylation [19]. To investigate whether the changes in the acetylation status of FOXO1, FOXO3a, and FOXO6 were associated with SIRT1, the HG + SRT1720 group was examined. The results presented in Fig. 3B showed that the HG + SRT1720 group inhibited the expression of Ac-FOXO1 and Ac-FOXO3a proteins compared to the NG group (*p* < 0.05). Moreover, a COIP assay demonstrated that SIRT1 interacted with FOXO1 and FOXO3a (Fig. 3C). To assess cell viability and proliferation, a CCK-8 assay and EDU staining were performed, respectively. The NG, HG, HG + OE-Apelin, HG + OE-NC, and HG + OE-Apelin + EX527 subgroups were examined. As shown in Fig. 3D, the HG + OE-Apelin + EX527 group exhibited enhanced cell viability compared to the HG + OE-Apelin group. Similarly, the number of EDU-positive cells was significantly higher in the HG group, while Apelin overexpression in the HG group reduced the proportion of EDU-positive cells (*p* < 0.05, Fig. 3E). The addition of EX527 dramatically promoted cell proliferation. The levels of the proliferation markers PCNA and CyclinD1 were upregulated in HG group, and Apelin overexpression abolished these differences (*p* < 0.05). Moreover, the addition of EX527 showed increased PCNA and CyclinD1 expression in cells (*p* < 0.05, Fig. 3F). SIRT1 expression was downregulated, while α-SMA, fibronectin, and collagen I were upregulated in HG group (*p* < 0.05, Fig. 3G), and Apelin overexpression had the reversed effects. Furthermore, the HG + OE-Apelin + EX527 group showed decreased SIRT1 level and enhanced α-SMA, fibronectin, and collagen I levels compared to the HG + OE-Apelin group (*p* < 0.05). The acetylation levels of FOXO1 and FOXO3a were upregulated by HG treatment (Fig. 3H), and Apelin overexpression abolished these differences. Moreover, the HG + OE-Apelin + EX527 group showed increased acetylation levels of FOXO1 and FOXO3a compared to the HG + OE-Apelin group (*p* < 0.05). As FOXO1 and FOXO3a phosphorylation and nucleocytoplasmic shuttling is related to their acetylation status, our further studies presented in Fig. 3I and J demonstrated that high glucose increased phosphorylation levels of FOXO1 and FOXO3a, which can be partially reversed by a SIRT1 activator. Under high glucose conditions, FOXO1 and FOXO3a proteins are predominantly localized in the cytoplasm, whereas treatment with the SIRT1 activator decreases the cytoplasmic levels and increases the nuclear levels of FOXO1 and FOXO3a. All of the data indicates that Apelin can enhance the expression of SIRT1, which further affects FOXO deacetylation.

### RUNX3 transcription activated apelin expression

**Fig. 4:**
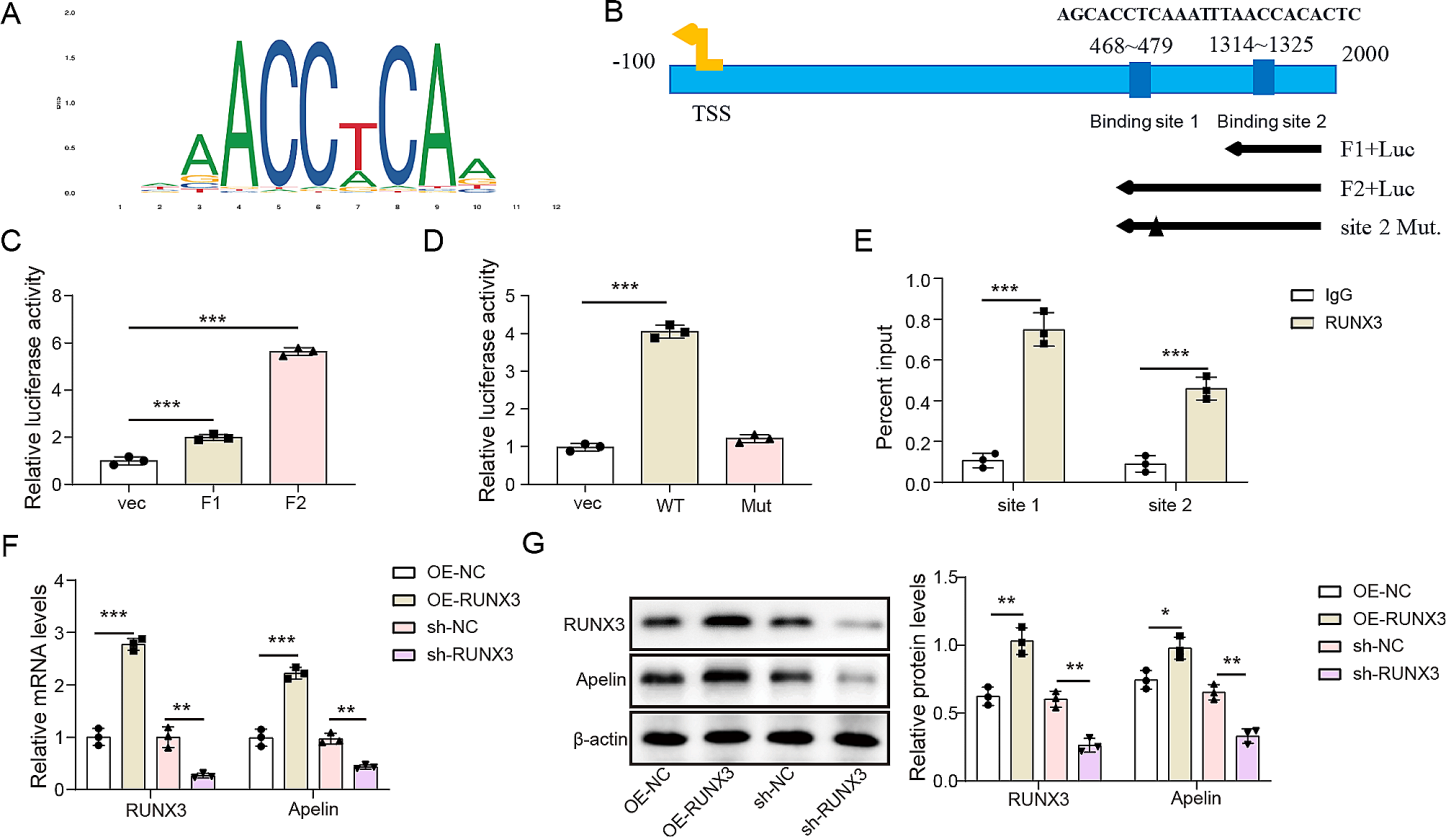
RUNX3 transcription activated Apelin expression. (**A**) The potential binding relations between RUNX3 and Apelin predicted by JASPAR. (**B**) The binding regulatory map. (**C**) Identification of the binding relationship between RUNX3 and the full-length binding site and the Site 2 by dual luciferase reporter assay. (**D**) Identification of the binding relationship between RUNX3 and the Site 1 mutant, Site 2 mutant, Site 1 + Site 2 double mutant sequences, and the wild-type sequence by dual luciferase reporter assay. (**E**) ChIP assay further examined the binding relations between RUNX3 and Apelin promoter. (**F**) The relative mRNA expression of RUNX3 and Apelin detected by RT-qPCR. (G) The protein expression of RUNX3 and Apelin determined using western blotting. *p < 0.05, **p < 0.01, ***p < 0.001. high glucose: HG; normal glucose: NG; overexpression: OE

RUNX3 plays a critical role in binding to DNA promoters and regulating diverse biological processes, however, its relationship with Apelin has not been investigated [24]. Here, the binding of RUNX3 to the Apelin promoter sequence 1 was predicted using the JASPAR database. Two potential RUNX3 binding consensus sequences were identified in the Apelin promoter, as shown in Fig. 4A. The binding regulatory map was constructed using full-length (F2) and truncated (F1) Apelin promoters, as well as the Apelin site 1 mutant promoter (Fig. 4B). To verify the targeting of RUNX3 to the Apelin promoter, a dual luciferase reporter results presented in Fig. 4C showed that the reporter genes containing site 1 (F2 group) exhibited high transcriptional activity than F1 group (*p* < 0.05). Luciferase activity of site 1 motif was dramatically reduced in the mutant (*p* < 0.05, Fig. 4D). Furthermore, the ChIP results showed that RUNX3 was enriched at both the site 1 and site 2 fragments of the Apelin promoter. (Fig. 4E). Overexpression of RUNX3 activated Apelin, while interference with RUNX3 expression inhibited Apelin (*p* < 0.05, Fig. 4F and G). These findings suggest that RUNX3 may bind to the promoter and regulate the expression of Apelin.

### Interfering RUNX3 reversed Apelin’s effects on MC proliferation through SIRT1/FOXO signaling pathway

**Fig. 5:**
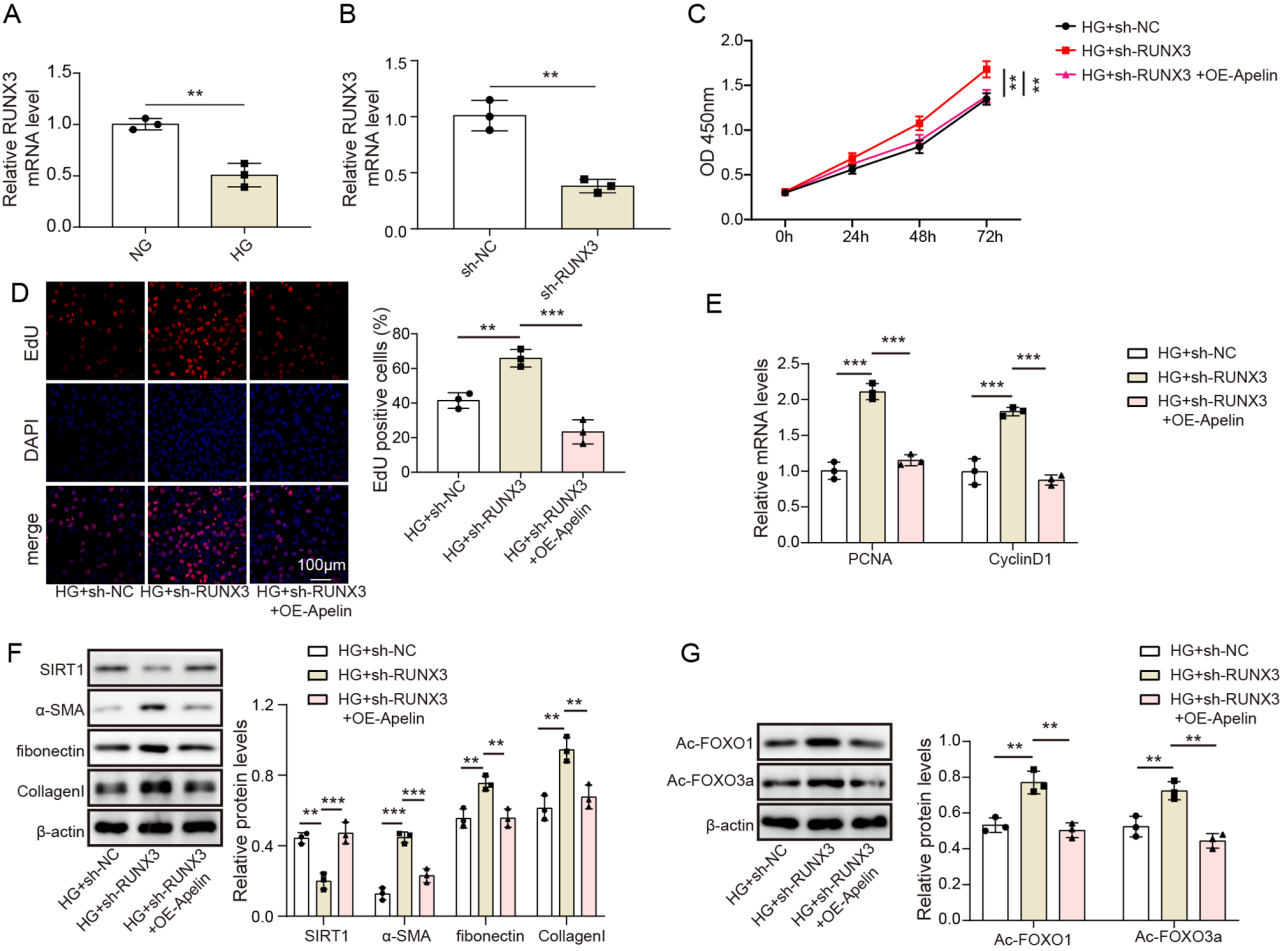
Interfering RUNX3 reversed the inhibitory effects of Apelin on MCs proliferation and fibrosis in DN through SIRT1/FOXO signaling pathway. (**A**) The relative mRNA expression of RUNX3 detected by RT-qPCR. (**B**) Cell viability evaluated by CCK8 assay. (**C**) Cell proliferation determined using EDU staining. (**D**) The relative mRNA expression of PCNA and CyclinD1 detected by RT-qPCR. (**E**) The protein expression of SIRT1, α-SMA, fibronectin, and collagen I determined using western blotting. (**F**) The acetylation status of FOXO1 and FOXO3a assessed by western blotting. *p < 0.05, **p < 0.01, ***p < 0.001. high glucose: HG; normal glucose: NG; overexpression: OE

Figure 5A showed the change in RUNX3 in HG-stimulated MC. As compared to NG group, HG group developed the downregulated RUNX3 in MC. Moreover, we demonstrated RUNX3 and SIRT1 were significantly decreased in mice with DN and HG-stimulated MC (Figure S3&S4), further suggesting DN could inhibit expressions of RUNX3 and SIRT1. To explore the role of RUNX3 on the proliferation and fibrosis of MCs in DN, the level of RUNX3 was determined by RT-qPCR, and successful transfection of sh-RUNX3 was confirmed as depicted in Fig. 5B. As illustrated in Fig. 5C, inhibition of RUNX3 resulted in increased cell viability, while overexpression of Apelin countered the effect of RUNX3 interference (*p* < 0.05). Cell proliferation was evaluated by EDU assay, as depicted in Fig. 5D. Transfection of sh-RUNX3 promoted cell proliferation, and transfection of OE-Apelin vectors had reversed effects (*p* < 0.05). Inhibiting RUNX3 increased PCNA and CyclinD1 expression levels in cells (Fig. 5E), and overexpression of Apelin counteracted the effect of RUNX3 interference (*p* < 0.05). HG + sh-RUNX3 increased the expression levels of α-SMA, fibronectin, and collagen I in cells compared with the HG + sh-NC group, and decreased SIRT1 expression (*p* < 0.05, Fig. 5F). However, the results were reversed in HG + sh-RUNX3 + OE-Apelin group. The levels of acetylation of FOXO1 and FOXO3a were also detected by western blotting assay (Fig. 5G). HG + sh-RUNX3 increased the acetylation levels of FOXO1 and FOXO3a in cells, and the effect of sh-RUNX3 was eliminated by Apelin overexpression (*p* < 0.05). It can be concluded that RUNX3 was important for regulating Apelin and SIRT1/FOXO in DN progression.

### Apelin overexpression and RUNX3 interference impacted the DN and SIRT1/FOXO signaling pathways

**Fig. 6:**
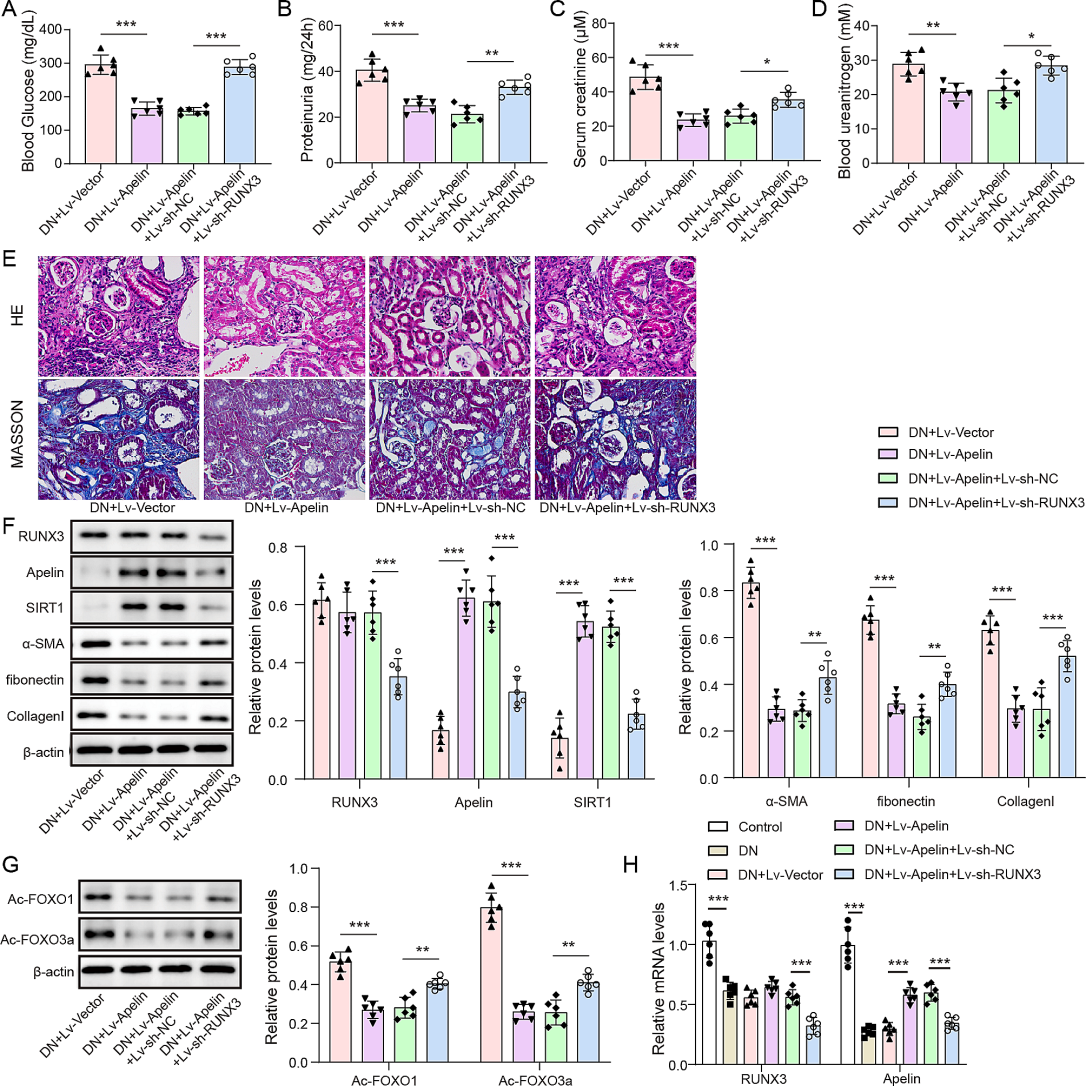
Interfering RUNX3 reversed the ameliorated DN progression by targeting Apelin-regulated SIRT1/FOXO signaling pathway (*n* = 6 in each group). (**A**) The blood glucose level. (**B**) The 24-hour proteinuria level. (**C**) The serum creatinine level. (**D**) The blood urea nitrogen level. (**E**) The H&E and Masson staining revealed the kidney structural changes (400X). (**F**) The protein expression of RUNX3, Apelin, SIRT1, α-SMA, fibronectin, and collagen I determined using western blotting. (**G**) The acetylation status of FOXO1 and FOXO3a assessed by western blotting. (**H**) The relative mRNA expression of RUNX3 and Apelin detected by RT-qPCR. *p < 0.05, **p < 0.01, ***p < 0.001. diabetic nephropathy: DN; hematoxylin and eosin: H&E; high glucose: lentivirus: Lv

To validate whether the regulatory mechanisms of Apelin in the DN mouse model corresponded to the cellular model, we divided the mice into 6 groups (*n* = 6 per group): control, DN, DN + LV-Vector, DN + Lv-Apelin, DN + LV-Apelin + LV-sh-NC and DN + Lv-Apelin + Lv-sh-RUNX3. Glucose levels, 24-hour proteinuria, and serum creatinine were quantified to evaluate renal function in mice (Fig. 6A-D). These indices were dramatically enhanced in the DN group, indicating kidney damage. Overexpression of Apelin attenuated renal injury, while knockdown of RUNX3 expression reversed the improvement in renal injury caused by Apelin overexpression (*p* < 0.05). H&E and Masson staining were conducted to evaluate kidney pathology in mice (Fig. 6E). Overexpression of Apelin improved renal structure and interstitial fibrosis, reduced the degree of mesangial substrate thickening, vacuolation of renal tubular epithelial cells and interstitial inflammatory cell infiltration, and knockdown of RUNX3 expression reversed the renal injury improved by overexpression of Apelin. The western blotting data revealed that knockdown of RUNX3 expression reversed the effects of Apelin overexpression on fibrotic factors including α-SMA, fibronectin, and collagen I levels (Fig. 6F). Moreover, in kidney tissues, the acetylation of FOXO1 and FOXO3a were promoted in the DN group, while the level of RUNX3 was reduced (*p* < 0.05, Figure  S5&S6). Overexpression of Apelin inhibited acetylation of FOXO1 and FOXO3a in kidney tissues and promoted the expression levels of Apelin and SIRT1 (*p* < 0.05, Fig. 6G and H). However, knockdown of RUNX3 expression reversed the effect of overexpression of Apelin (Fig. 6G and H).

## Discussion

In the present study, we found that Apelin level was decreased in both STZ-induced DN mice models and HG-treated DN cell models. Our results showed that Apelin overexpression reduced the levels of α-SMA, fibronectin, and collagen I, as well as decreased the degree of mesangial substrate thickening, vacuolation of renal tubular epithelial cells and interstitial inflammatory cell infiltration. Mesangial expansion caused by mesangial cell proliferation and extracellular matrix deposition is an important cause of glomerulosclerosis. Alpha SMA reflects the mesangial cell trans differentiation and can increase the contractile force of glomerular mesangial cells and lead to glomerular hemodynamics changes, eventually lead to glomerular sclerosis. α-SMA can also induce the secretion of ECM such as FN and collagen I and promote glomerulosclerosis [32, 33]. The inhibition of mesangial cell proliferation and fibrosis factor secretion by Apelin is beneficial to improve mesangial cell-centered glomerulosclerosis in DN. Regarding the role of Apelin in DN, Chen et al. reported that Apelin-13 treatment decreased diabetes-induced glomerular filtration rate, proteinuria and renal inflammation [34]. The descreased Apelin level was also suggested to be a potential biomarker for diabetic nephropathy in patients with type 2 diabetes [15]. These findings indicate that Apelin has anti renal fibrosis effects and might be a molecular treatment target for DN, providing potential molecular targets for clinical treatments of DN.

The SIRT1/FOXO signaling pathways is demonstrated to participate in multiple crucial mechanisms, which included cell apoptosis, oxidative stress resistance, and tumor progression [35]. This study firstly found that Apelin could increase the HG-induced reduction in SIRT1 levels and reduced the expression of Ac-FOXO1 and Ac-FOXO3a. Furthermore, inhibition of SIRT1 reversed the effects of Apelin on FOXO1/FOXO3a acetylation and promoted the progression of DN in vitro. Similar with our findings, Yang et al. showed that fucoxanthin increased SIRT1 level, reduced the acetylation of FOXO3a, and alleviated HG-induced renal fibrosis. However, inhibiting SIRT1 using EX257 reversed the effects of fucoxanthin [36]. However, in our study, Apelin is identified as an endogenous ligand with higher biocompatibility. Additionally, we found that SIRT1 specifically regulates both FOXO3a and FOXO1, extending the findings of Yang et al. The phosphorylation of FOXO1 and FOXO3a by Akt promotes their cytoplasmic translocation, while acetylation increases their sensitivity to Akt phosphorylation [37]. Our results show that high glucose induces Akt-mediated phosphorylation and cytoplasmic accumulation of FOXO1/3a in mesangial cells. This intriguing result may be related to the cross-talk of Akt mediated phosphorylation and acetylation. Acetylation also inhibits the DNA binding and nuclear localization of these proteins [38]. Notably, SIRT1 deacetylates FOXO1/3a, reducing their phosphorylation by Akt and promoting their nuclear retention. By restoring the nuclear localization and transcriptional activity of FOXO1/3a, SIRT1 may facilitate the expression of target genes that protect against high glucose-induced mesangial cell injury in diabetic nephropathy. Thus, modulating the post-translational modifications and subcellular distribution of FOXO proteins emerges as a potential therapeutic strategy.

RUNX3, which belongs to the RUNX family with a highly conserved DNA sequence, interacts with proteins that are crucial for regulating various biological processes. In the present study, we newly identified the interactions between RUNX3 and activation of Apelin. Depletion of RUNX3 significantly reversed the protective effects of Apelin in DN. Moreover, RUNX3 was found to be a crucial gene for predicting progressive interstitial fibrosis and tubular atrophy [39], indicating the potential role of RUNX3 in treating DN through Apelin. The above studies indicate that RUNX3 possesses transcriptional factor activity. In 2023, Huang et al. [40] pointed out that TGF-β/Smad signaling pathway is the upstream pathway mediating RUNX3 nuclear import in tumor models, and nuclear RUNX3 has a negative regulatory mechanism on TGF-β/Smad pathway. In addition, some studies have found that Apelin can inhibit the expression of TGF-β/smad pathway to improve fibrosis in DN model [40–42]. However, the regulatory relationship between RUNX3 and Apelin is unknown. Our study confirmed that RUNX3 has a transcriptional activation effect on Apelin, suggesting that the RUNX3/Apelin pathway may have crosstalk with the TGF-β/smad pathway, which is a recognized central signaling pathway in anti-organ fibrosis studies. Apelin inhibits the expression of TGF-β/smad, and TGF-β/smad may regulate RUNX3 to enter the nucleus, thereby implementing the negative feedback regulation of Apelin against renal fibrosis. TGF-β antagonists have been experimentally used as antifibrotic therapies, but with poor efficacy. Enhance the activity of endogenous anti fibrosis molecular Apelin may provide a new train of thought for anti fibrosis treatment. Moreover, in the in vitro model, as shown in Results Fig. 6E and F, the anti-proliferative and anti-fibrotic effects of Apelin overexpression could only be partially reversed by RUNX3 interference, suggesting that other upstream activation pathways of Apelin may exist besides RUNX3. All these provide new ideas for our follow-up research.

Our research is the first to demonstrate the transcriptional regulatory mechanism of RUNX3 on Apelin expression. Although this study confirmed the upstream and downstream regulatory factors of Apelin, the specific mechanism of Apelin’s effect on SIRT1 could not be deeply explored. SIRT1 improves the affinity between FOXO proteins and DNA by inducing FOXO protein deacetylation, thereby enhancing their DNA binding and promoting retention in the nucleus. This interaction helps activate target gene transcription and promotes gene expression. In conclusion, the SIRT1/FOXO pathway plays a crucial role in mediating gene transcription and expression. Therefore, further studies on the specific mechanism of Apelin’s regulation of SIRT1 will help to refine our understanding of Apelin’s anti-cell proliferation and anti-fibrosis mechanisms.

In summary, our study demonstrated that RUNX3 activated Apelin and regulated the SIRT1/FOXO signaling, contributing to the inhibition of cell proliferation and fibrosis in DN. Our findings provide important insights for the molecular role of Apelin and the involvement of RUNX3 in DN pathogenesis. However, it should be noted that our experiments utilized cell lines and an animal model, which may not fully recapitulate the human clinical condition and lack the functions and mechanisms of Apelin deficiency in MCs. Further studies using more physiologically relevant models are needed to validate these results. Nonetheless, clinical investigations are warranted to explore the therapeutic potential of targeting Apelin and RUNX3 for diabetic nephropathy. Apelin is a potential therapeutic target for endogenous anti-renal damage and anti-fibrosis in diabetic nephropathy. RUNX3 is expected to be an endogenous intervention target for Apelin deletion-related diseases.

### Electronic supplementary material

Below is the link to the electronic supplementary material.

Supplementary Material 1: Figure S1. The expression of Apelin in kidney tissues detected by immunofluorescence (scale: 50 mm; Red: Apelin; Blue: nucleus).

Supplementary Material 2: Figure S2. Apelin expression when cells are transduced with Apelin overexpression vector under normal glucose conditions. (**p < 0.01)

Supplementary Material 3: Figure S3. The changes in RUNX3 and SIRT1 in sham and DN group detected by immunofluorescence (scale: 50 mm; Green: RUNX3; Red: SIRT1; Blue: nucleus).

Supplementary Material 4: Figure S4. The changes in RUNX3 and SIRT1 in NG and HG group detected by western blot.

Supplementary Material 5: Figure S5. The acetylation status of FOXO1 and FOXO3a assessed by western blotting (***P < 0.001).

Supplementary Material 6: Figure S6. The changes in RUNX3 and SIRT1 in control and DN group detected by western blot (***P < 0.001).

## Data Availability

All data generated or analyzed during this study are included in this article. The datasets used and/or analyzed during the current study are available from the corresponding author on reasonable request.

## Abbreviations

H&E: hematoxylin and eosin
HG: high glucose
Lv: lentivirus
MCs: mesangial cells
MGECs: mouse glomerular endothelial cells
NG: normal glucose
OE: overexpression
RT-qPCR: real-time quantitative PCR
RUNX: runt-related transcription factors
SD: standard deviation
TGF-β1: transforming growth factor-β1
