# Supplementary figures and images for: RUNX3-activated apelin signaling inhibits cell proliferation and fibrosis in diabetic nephropathy by regulation of the SIRT1/FOXO pathway
Source: Diabetol Metab Syndr. 2024 Jul 17;16:167. doi: 10.1186/s13098-024-01393-x (PMC11253400; doi:10.1186/s13098-024-01393-x)

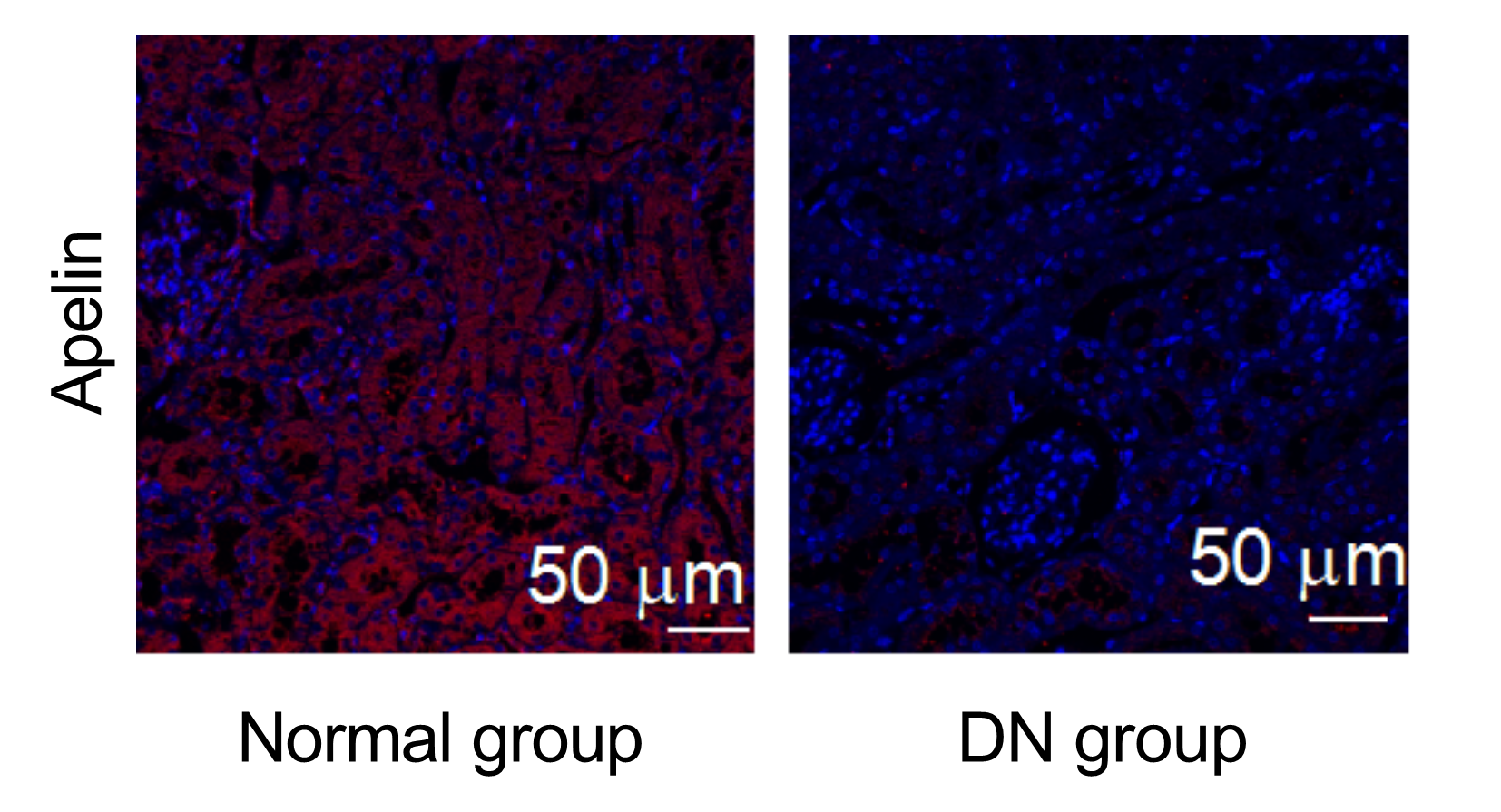

Supplement: Supplementary file 1 — Supplementary Material 1: Figure S1. The expression of Apelin in kidney tissues detected by immunofluorescence (scale: 50 mm; Red: Apelin; Blue: nucleus). [file 13098_2024_1393_MOESM1_ESM.png]

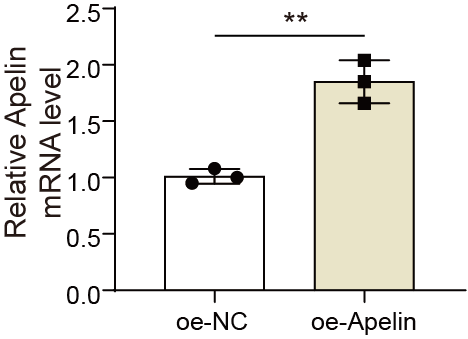

Supplement: Supplementary file 2 — Supplementary Material 2: Figure S2. Apelin expression when cells are transduced with Apelin overexpression vector under normal glucose conditions. (**p < 0.01) [file 13098_2024_1393_MOESM2_ESM.png]

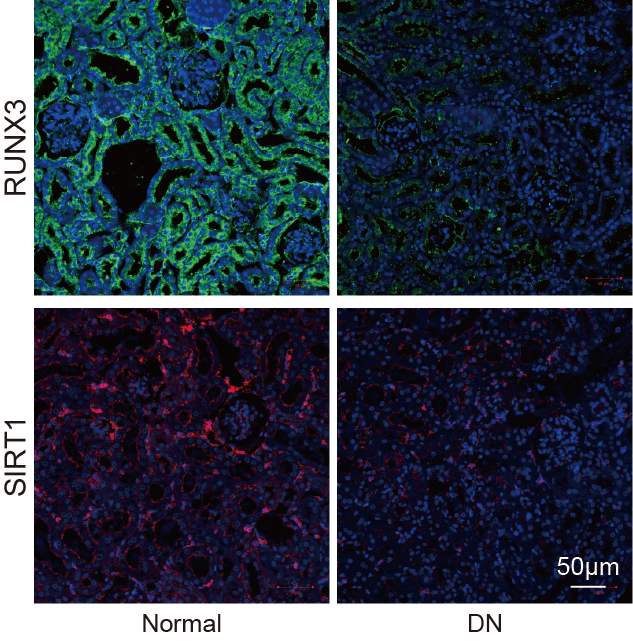

Supplement: Supplementary file 3 — Supplementary Material 3: Figure S3. The changes in RUNX3 and SIRT1 in sham and DN group detected by immunofluorescence (scale: 50 mm; Green: RUNX3; Red: SIRT1; Blue: nucleus). [file 13098_2024_1393_MOESM3_ESM.png]

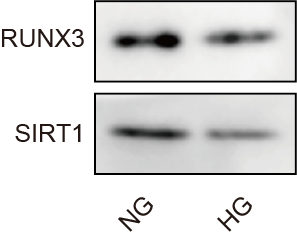

Supplement: Supplementary file 4 — Supplementary Material 4: Figure S4. The changes in RUNX3 and SIRT1 in NG and HG group detected by western blot. [file 13098_2024_1393_MOESM4_ESM.png]

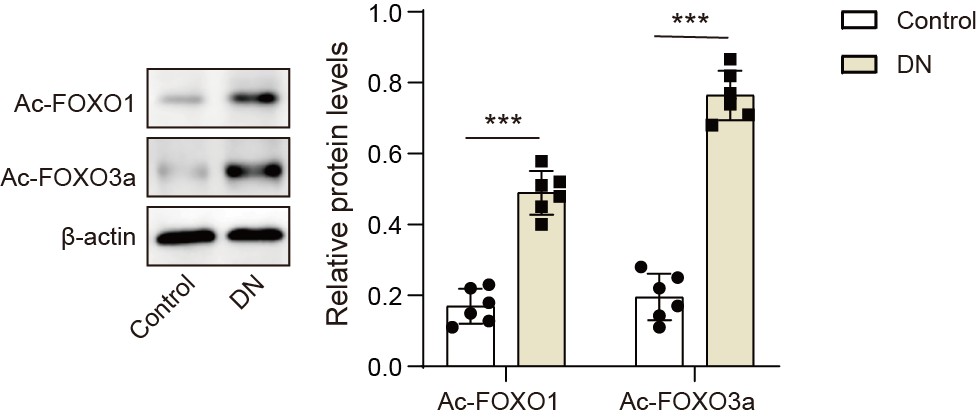

Supplement: Supplementary file 5 — Supplementary Material 5: Figure S5. The acetylation status of FOXO1 and FOXO3a assessed by western blotting (***P < 0.001). [file 13098_2024_1393_MOESM5_ESM.png]

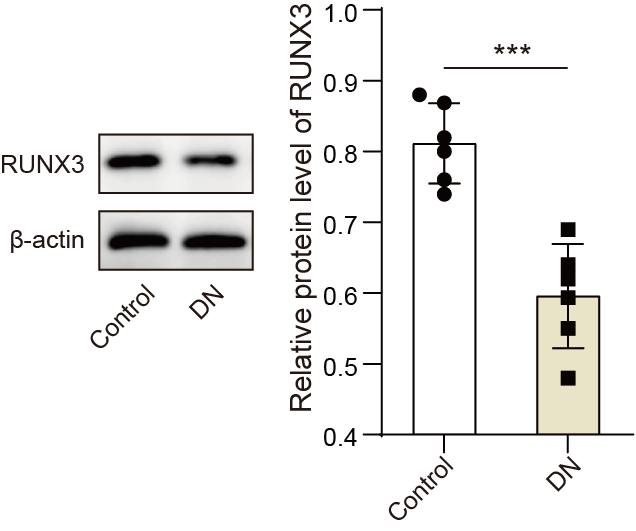

Supplement: Supplementary file 6 — Supplementary Material 6: Figure S6. The changes in RUNX3 and SIRT1 in control and DN group detected by western blot (***P < 0.001). [file 13098_2024_1393_MOESM6_ESM.png]
